# Metabolic changes and propensity for inflammation, fibrosis, and cancer in livers of mice lacking lysosomal acid lipase

**DOI:** 10.1016/j.jlr.2023.100427

**Published:** 2023-08-16

**Authors:** Ivan Bradić, Laura Liesinger, Katharina B. Kuentzel, Nemanja Vujić, Michael Trauner, Ruth Birner-Gruenberger, Dagmar Kratky

**Affiliations:** 1 Division of Molecular Biology and Biochemistry, Gottfried Schatz Research Center, Medical University of Graz, Graz, Austria; 2 Institute of Chemical Technologies and Analytics, TU Wien, Vienna, Austria; 3 Division of Gastroenterology and Hepatology, Department of Internal Medicine III, Medical University of Vienna, Vienna, Austria; 4 BioTechMed-Graz, Graz, Austria; 5 Diagnostic and Research Institute of Pathology, Medical University of Graz, Graz, Austria

**Keywords:** lysosomal storage disorder, lipid metabolism, cholesterol, non-alcoholic fatty liver disease, lipase/lysosomal, lipids, liver, proteomics, cholesterol ester storage disease

## Abstract

Lysosomal acid lipase (LAL) is the sole lysosomal enzyme responsible for the degradation of cholesteryl esters and triacylglycerols at acidic pH. Impaired LAL activity leads to LAL deficiency (LAL-D), a severe and fatal disease characterized by ectopic lysosomal lipid accumulation. Reduced LAL activity also contributes to the development and progression of non-alcoholic fatty liver disease (NAFLD). To advance our understanding of LAL-related liver pathologies, we performed comprehensive proteomic profiling of livers from mice with systemic genetic loss of LAL (*Lal−/−*) and from mice with hepatocyte-specific LAL-D (*hepLal−/−*). *Lal−/−* mice exhibited drastic proteome alterations, including dysregulation of multiple proteins related to metabolism, inflammation, liver fibrosis, and cancer. Global loss of LAL activity impaired both acidic and neutral lipase activities and resulted in hepatic lipid accumulation, indicating a complete metabolic shift in *Lal−/−* livers. Hepatic inflammation and immune cell infiltration were evident, with numerous upregulated inflammation-related gene ontology biological process terms. In contrast, both young and mature *hepLal−/−* mice displayed only minor changes in the liver proteome, suggesting that loss of LAL solely in hepatocytes does not phenocopy metabolic alterations observed in mice globally lacking LAL. These findings provide valuable insights into the mechanisms underlying liver dysfunction in LAL-D and may help in understanding why decreased LAL activity contributes to NAFLD. Our study highlights the importance of LAL in maintaining liver homeostasis and demonstrates the drastic consequences of its global deficiency on the liver proteome and liver function.

## Graphical abstract

**Figure:**
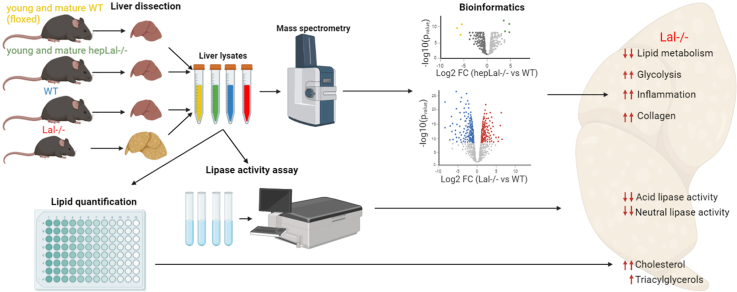

Lysosomal acid lipase (LAL) is crucial in the degradation of cholesteryl esters (CE) and triacylglycerols (TG) delivered to lysosomes either by endocytosis of lipoproteins (1) or by autophagy (2). Its metabolic relevance is underscored by the fact that patients with loss-of-function mutations in the LAL-encoding *LIPA* gene (3, 4) suffer from LAL deficiency (LAL-D; MIM#278000), a rare autosomal recessive disorder with an estimated prevalence of 1 per 177,000 individuals (3, 4). A complete loss of LAL activity leads to the development of early-onset LAL-D (previously known as Wolman disease), a severe condition that most patients succumb to within the first year of life (5, 6). Late-onset LAL-D (previously known as CE storage disease) is a less grave form with variable age of onset and severity in which up to 12% of normal LAL activity is preserved (7). The hallmark of LAL-D is an ectopic accumulation of CE and TG (8, 9), most prevalent in the liver and small intestine, impairing whole-body lipid metabolism (7). While multiple factors, including malabsorption, cachexia, and liver disease, contribute to the lethal outcomes in patients with early-onset LAL-D, late-onset LAL-D deaths are mostly due to liver failure and/or accelerated atherosclerosis (3). In addition to LAL-D, LAL may also play a role in the development of non-alcoholic fatty liver disease (NAFLD) and has been proposed as a potential blood biomarker of NAFLD severity (10, 11).

The function of LAL in lipid metabolism has been intensively studied in Lal-deficient (*Lal−/−*) mice (12, 13). These animals show massive ectopic accumulation of CE and TG in different organs, including the liver, intestine, and spleen, while suffering from lipodystrophy (12, 13). Furthermore, *Lal−/−* mice have increased circulating total cholesterol (TC) levels, with elevated LDL- and decreased HDL-cholesterol (14). Notably, these mice exhibit markedly reduced body weight compared to their WT littermates, highlighting the impact of LAL-D on overall metabolic health (14). In addition to *Lal−/−* mice, the characterization of hepatocyte-specific *Lal−/−* (*hepLal−/−*) mice has deepened our understanding of the role of LAL in liver metabolism (15, 16), as *hepLal−/−* mice display CE accumulation confined to the liver (15). When fed a high-fat/high-cholesterol diet (HF/HCD), hepatocyte-specific LAL-D is sufficient to alter whole-body lipid and energy homeostasis in mice (15). Despite being protected from diet-induced obesity, these mice have increased hepatic inflammation, suggesting a complex interplay between LAL-D and hepatic lipid homeostasis (15).

To unbiasedly investigate changes in the liver proteome of *Lal−/−* and *hepLal−/−* mice and to systematically identify alterations in hepatic protein expression and metabolic pathways upon LAL-D, we utilized mass spectrometry-based proteomics analysis. We observed significant proteome changes in the livers of *Lal−/−* mice, with proteins related to inflammation, cholesterol metabolism, glycolysis, and cancer development being highly upregulated. In contrast, proteins related to fatty acid (FA) metabolism were downregulated in the livers of *Lal−/−* mice. The data from this study may contribute substantially to the understanding of the pathogenesis of LAL-D.

## Materials and methods

### Animals

Mice were housed in a clean and temperature-controlled (22°C ± 1°C) environment with unlimited access to a chow diet (Altromin 1324, Lage, Germany) and water on a regular 12-h light/12-h dark cycle. After a 4 h fasting period, mice were gavaged with 200 μl of olive oil, and organs and blood were collected 2 h post-gavage. Female WT and *Lal−/−* mice (12) were sacrificed between 11 and 15 weeks of age. Female *hepLal−/−* mice (15) and their corresponding Lal^flox/flox^ littermates were used in two different age groups. Mice aged between 9 and 11 weeks were referred to as young *hepLal−/−* and young WT, respectively, whereas mice aged between 50 and 60 weeks were referred to as mature *hepLal−/−* and mature WT, respectively. A cohort of WT and *Lal−/−* mice were sacrificed after 4 h of fasting without gavage and designated as WT fasted and *Lal−/−* fasted, respectively. All animal experiments were performed according to the European Directive 2010/63/EU in compliance with national laws and approved by the Austrian Federal Ministry of Education, Science and Research, Vienna, Austria (BMWFW-66.010/0109-WF/V/3b/2015, 2020-0.129.904).

### Tissue lipid extraction and quantification

Half of the largest liver lobe was lysed on ice in lysis buffer by 6 × 10 s sonication. Lysates were centrifuged for 10 min at 20,000 *g* and 4°C, and protein concentrations in the supernatants were measured using the DC™ Protein Assay Kit (Bio-Rad Laboratories, Hercules, CA). Lipids were extracted from the lysates containing 1 mg of protein by the Folch method. TG and TC concentrations were determined using enzymatic kits (DiaSys, Holzheim, Germany) according to the manufacturer's guidelines.

### Lipase activity assays

Neutral (pH 7) and acidic (pH 4.5) CE hydrolase (CEH) and TG hydrolase (TGH) activities in liver lysates were measured using radioactively labeled substrates as previously described (17) with minor modifications. Briefly, half of the largest liver lobe was lysed in lysis buffer supplemented with 1 mM dithiothreitol and a protease inhibitor cocktail. After centrifugation, the supernatant was collected, and protein concentrations were determined as described earlier. To measure CEH activities, the substrate contained 200 μM cholesteryl oleate and 0.04 μCi cholesteryl [1-^14^C]-oleate (Amersham Biosciences) per sample, whereas TGH activities were determined with a substrate containing 300 μM triolein and 0.5 μCi [9,10-^3^H(N)]-triolein (PerkinElmer) per sample. The substrates for the CEH activity assay at pH 4.5 and 7 and for the TGH activity assay at pH 4.5 were emulsified in 455 μM mixed micelles of phosphatidylcholine and phosphatidylinositol (3:1). The substrate for the TGH activity assay at pH 7 was emulsified in 45 μM of above-mentioned micelles. The reconstituted substrates were mixed with 50 μg of liver lysate proteins diluted in citrate buffer for assays at pH 4.5 and in potassium phosphate buffer for assays at pH 7. The rest of the assay was performed as previously described (18), and activities were calculated from the release of FA (19).

### Plasma lipid quantification

Freshly drawn EDTA blood was centrifuged for 7 min at 5,200 *g* and 4°C, and the supernatant was collected as plasma. TG and TC concentrations were determined with enzymatic kits as described earlier.

### Sample preparation and processing for proteomics analysis

Liver tissues were lysed in 100 mM Tris-HCl (pH 8.5) containing 1% SDS, 10 mM tris(2-carboxyethyl)phosphine (TCEP), and 40 mM chloroacetamide using a Miccra homogenizer in combination with a Pico tool suitable for a volume range of 0.1–10 ml (Miccra, Heitersheim, Germany). Samples were then reduced and alkylated for 10 min at 95°C and centrifuged for 5 min at 7,000 *g* and 4°C to remove cell debris, followed by protein estimation using the Pierce™ BCA Protein Assay. Fifty micrograms of each sample was precipitated with acetone, dissolved in 25% 2,2,2-trifluoroethanol (TFE)/50 mM Tris-HCl (pH 8.5), diluted with 50 mM ammonium bicarbonate to <10% TFE and digested by adding Promega Trypsin/LysC Mix in a 25:1 ratio and shaking overnight at 550 rpm and 37°C. Four micrograms of the resulting peptide solution was acidified to a final concentration of 1% trifluoroacetic acid and desalted using in-house-made stage tips with styrene-divinylbenzene - reversed phase sulfonate (SDB-RPS) as material.

### LC-MS/MS

Peptides were separated on the Ultimate 3000 RSLC Nano Dionex system equipped with an IonOpticks Aurora Series UHPLC C18 column (250 mm × 75 μm, 1.6 μm) (IonOpticks); 0.1% formic acid in water was used as solvent A and acetonitrile containing 0.1% formic acid was used as solvent B. The total LC-MS/MS run time per sample was 136.5 min with the following gradient: 0–5.5 min: 2% B; 5.5–65.5 min: 2–17% B; 65.5–95.5 min: 17–25% B, 95.5–105.5 min: 25–37% B, 105.5–115.5 min: 37–95% B, 115.5–125.5 min: 95% B; 125.5–126.5 min: 95–2% B; 126.5–136.5 min: 2% B at a flow rate of 400 nl/min at 40°C. The timsTOF Pro mass spectrometer (Bruker Daltonics GmbH) was operated in positive mode with enabled trapped ion mobility spectrometry (TIMS) at 100% duty cycle (100 ms ramp time, 100–1,700 m/z mass range, 0.6–1.6 V s cm^−2^ ion mobility range). The source capillary voltage was set to 1,500 V and the dry gas flow to 3 l/min at 180°C. The scan mode was set to data-dependent acquisition parallel accumulation–serial fragmentation (DDA - PASEF) with 10 PASEF MS/MS scans per acquisition cycle (overall DDA cycle time 1.1 s).

### MS data processing

Protein identification and label-free quantification were performed by analyzing MS/MS data using MaxQuant 2.0.1.0 against the UniProt public database with the taxonomy Mus musculus and common contaminants (downloaded 2021/08/17, 17,219 sequences). Carbamidomethylation at Cys was used as a fixed modification, oxidation at Met was entered as a variable modification, and the following search criteria were used: trypsin, maximum number of missing cleavage sites: 2; search mode: MS/MS ion search with decoy database search included; precursor mass tolerance ± 10 ppm; product mass tolerance ± 40 ppm; acceptance parameters for identification: 1% PSM FDR and 1% protein FDR. Label-free quantitation (LFQ), including the "match between runs" feature of MaxQuant and requiring a minimum of 2 ratio counts of quantified razor plus unique peptides, was performed (20).

### Bioinformatics and statistical analysis of proteomics data

Data processing using protein group quantities was performed with Perseus software version 1.6.15.0 (21) and Jupyter Notebook using Python version 3.9. One young WT and one young *hepLal−/−* mouse liver were excluded from proteomic analysis due to fewer detected proteins. Intensities were log_2_ transformed to reduce the effects of outlier values and filtered for four valid values in at least one group. Missing intensities were replaced by random values taken from the Gaussian distribution of values using default parameters (width of 0.3, downshift of 1.8) to simulate a value for these low-abundant proteins. For principal component analysis (PCA), intensities were standardized (data mean = 0, data standard deviation = 1), and analysis was performed with Jupyter Notebook using the sklearn, seaborn, and bioinfokit packages. Two-sample t-tests followed by multiple testing correction by the permutation-based FDR method were used to identify altered protein groups. S0 = 2 for WT versus *Lal−/−*, S0 = 0.1 for young WT versus young *hepLal−/−* and mature WT versus mature *hepLal−/−*. Hierarchical clustering analysis was performed using Euclidean distance. Two-dimensional enrichment analysis on gene ontology biological process (GOBP) and gene ontology cellular component (GOCC) was performed using clusters of significantly upregulated and downregulated proteins from livers of WT and *Lal−/−* mice. Analysis was performed using Fisher's exact test, and significance was corrected by Benjamini–Hochberg with a false discovery rate (FDR) < 0.02. The enrichment factor represents the ratio between the number of proteins observed in a given term and the number of proteins expected in that term based on all proteins detected. For livers from young and mature WT and *hepLal*−/− mice, one-dimensional enrichment analysis (22) using protein log_2_-fold change values was performed on the Kyoto encyclopedia of genes and genomes (KEGG), and significance was corrected by Benjamini–Hochberg with FDR < 0.02. The enrichment score indicates whether the log_2_-fold change of proteins belonging to the specific annotation is larger or smaller than the distribution of the log_2_-fold change of all detected proteins. To count significantly changed proteins from WT and *Lal−/−* mice livers annotated to specific UniProt keyword terms, reviewed (Swiss-Prot) proteins annotated to certain keywords were used (supplemental Tables S1–S3).

### Statistical analysis

Data are presented as mean ± SD. Statistical significance between the groups was determined using GraphPad Prism 9.5.1 software. Comparison between two groups was performed using unpaired two-tailed Student's *t* test, and comparisons of multiple groups were analyzed by two-way ANOVA followed by Tukey's post hoc test. Significance levels were set as follows: ∗*P* < 0.05, ∗∗*P* ≤ 0.01, ∗∗∗*P* ≤ 0.001, and ∗∗∗∗*P* ≤ 0.0001 for comparison between different genotypes within the same group (WT vs. *Lal−/−*, WT fasted vs. *Lal−/−* fasted, young WT vs. young *hepLal−/−*, mature WT vs. mature *hepLal−/−*); ^#^*P* < 0.05, ^##^*P* ≤ 0.01, ^###^*P* ≤ 0.001, and ^####^*P* ≤ 0.0001 for comparison between the different conditions within the same genotype (WT fasted vs. WT, *Lal−/−* fasted vs. *Lal−/−*, young WT vs. mature WT, young *hepLal−/−* vs. mature *hepLal−/−*).

## Results

### Distinct changes in the liver proteome of *Lal−/−* mice

**Fig. 1:**
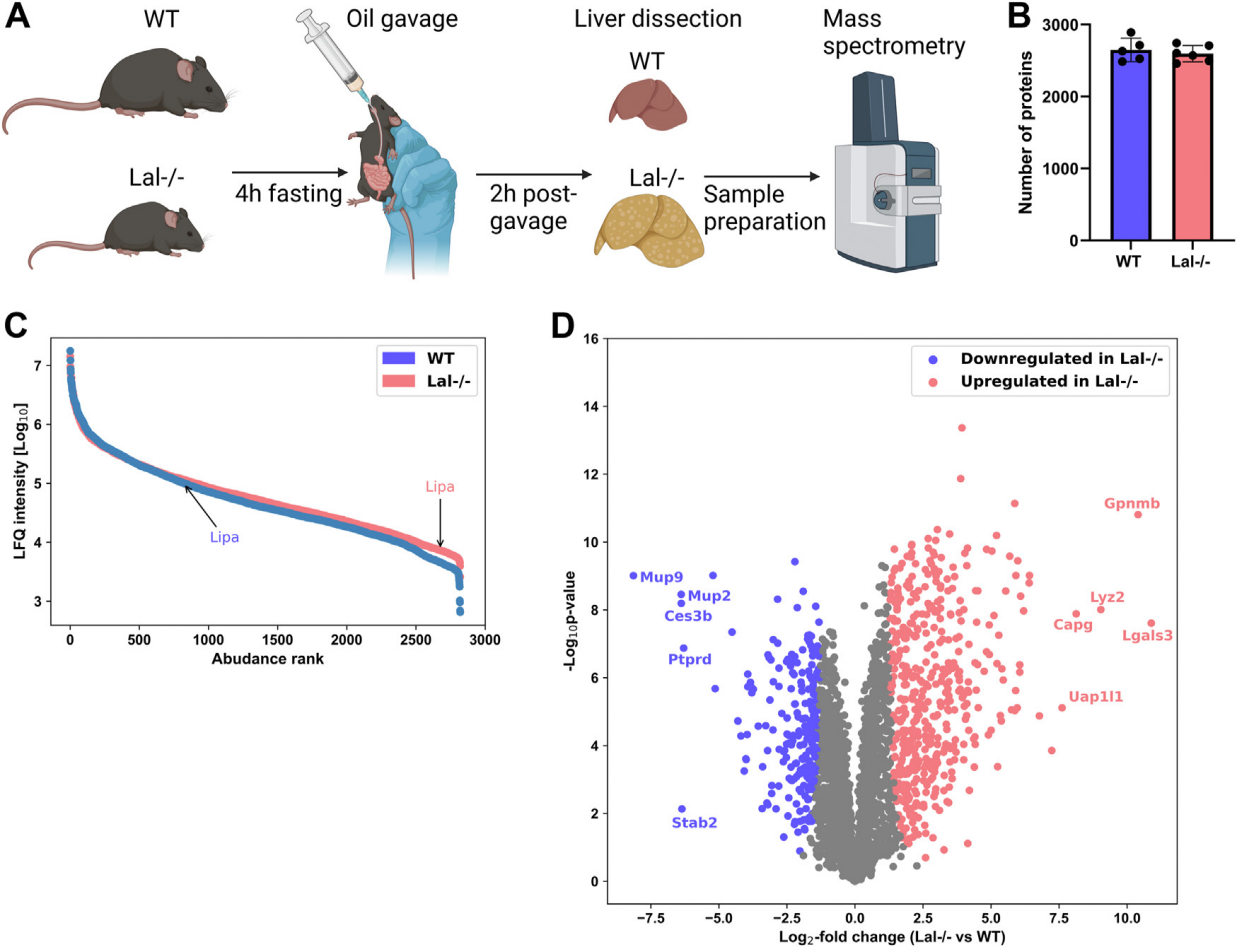
Significant proteome alterations in livers of Lal-deficient (*Lal−/−*) mice. A: Experimental setup of 4 h fasting of wild-type (WT) and *Lal−/−* mice followed by an oral oil bolus and sacrifice 2 h post-gavage. B: Quantified total proteome depth in wild-type (WT) and *Lal−/−* mice livers. C: Dynamic range of the liver proteome from WT and *Lal−/−* mice based on log_10_ of the mean intensity of relative label-free quantification (LFQ), ordered by rank of abundance. D: Volcano plot of the liver proteome showing 480 significantly upregulated and 234 significantly downregulated proteins in *Lal−/−* mice (FDR < 0.01, S0 = 2). Figures represent data from 5 WT and 6 *Lal−/−* mice. Data are presented as mean ± SD.

To obtain a broad overview of proteome alterations caused by LAL-D, we first performed proteomics analysis using livers of young (11–15 weeks of age) female *Lal−/−* and WT mice (Fig. 1A). In agreement with our previous studies (14, 18), the body weight of *Lal−/−* mice was significantly decreased compared with WT animals (supplemental Fig. S1A). The drastic remodeling of the liver is reflected in a more than 3-fold increase in the ratio of liver to body weight (supplemental Fig. S1B). By applying an FDR of 0.01, we identified an average of 2,649 and 2,596 proteins in WT and *Lal−/−* livers, respectively (Fig. 1B). After filtering for a minimum of four valid values in at least one genotype and imputing missing values (supplemental Fig. S1C), a total of 2,820 normally distributed proteins were quantified. The abundance of quantified proteins covered approximately four orders of magnitude (Fig. 1C). PCA revealed that 59.7% of the variance between *Lal−/−* and WT livers could be explained by the first component and 11.28% by the second component (supplemental Fig. S1D).

Of the 714 significantly changed proteins (FDR < 0.01, S0 = 2), 480 were upregulated and 234 were downregulated in *Lal−/−* livers (Fig. 1D). The five most upregulated proteins with the largest fold change were galectin-3 (LGALS3), transmembrane glycoprotein NMB (GPNMB), lysozyme C-2 (LYZ2), macrophage-capping protein (CAPG), and UDP-N-acetylhexosamine pyrophosphorylase-like protein 1 (UAP1L1) (Fig. 1D). The most downregulated proteins were major urinary protein 9 (MUP9), MUP2, carboxylesterase 3B (CES3B), stabilin-2 (STAB2), and receptor-type tyrosine-protein phosphatase delta (PTPRD) (Fig. 1D). The majority of these dysregulated proteins are related to metabolism or inflammation.

### Increased glucose metabolism and decreased lipid metabolism in LAL-D liver proteome

**Fig. 2:**
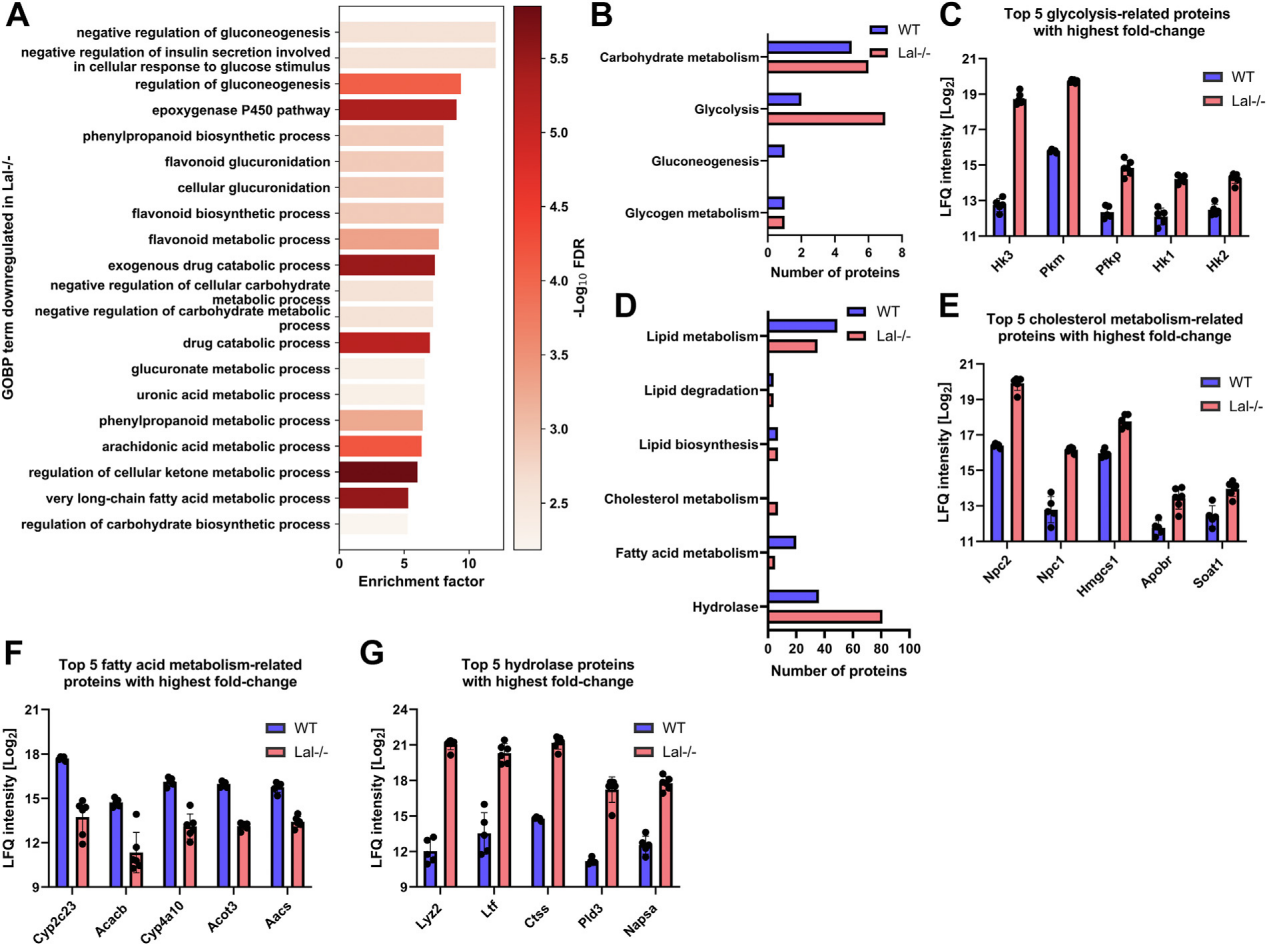
LAL deficiency impairs hepatic glucose and lipid metabolism. A: Top 20 gene ontology biological process (GOBP) terms downregulated in livers of *Lal−/−* mice. B: Significantly changed proteins from WT and *Lal−/−* livers annotated to selected carbohydrate metabolism-related UniProt keyword terms. C: Top five proteins with highest fold change annotated to *Glycolysis* UniProt keyword. D: Significantly changed proteins from WT and *Lal−/−* livers annotated to selected lipid metabolism-related UniProt keyword terms. Top five proteins with the highest fold change annotated to (E) *Cholesterol metabolism*, (F) *Fatty acid metabolism*, and (G) *Hydrolase* UniProt keywords. All figures represent data from 5 WT and 6 *Lal−/−* mice. Data are presented as mean or as mean ± SD.

Next, we performed two-dimensional enrichment analyses for GOBP terms to identify downregulated biological processes in the livers of *Lal−/−* mice. This systematic approach unveiled significant alterations in carbohydrate and lipid metabolic processes in the 20 most enriched GOBP terms (Fig. 2A). To further investigate metabolic dysregulation, we quantified the number of significantly up- or downregulated proteins in *Lal−/−* livers annotated with specific UniProt keywords. This analysis identified a higher number of significantly upregulated proteins associated with glycolysis in *Lal−/−* compared to WT mouse livers (Fig. 2B), including hexokinase 3 (HK3), pyruvate kinase (PKM), ATP-dependent 6-phosphofructokinase (platelet type; PFKP), HK1, and HK2 (Fig. 2C). Deregulation of these key enzymes of glycolysis suggests that *Lal−/−* mice are more dependent on glucose than WT mice. In addition, proteins related to cholesterol metabolism and hydrolases were upregulated, whereas proteins linked to lipid and FA metabolism were downregulated in the livers of *Lal−/−* mice (Fig. 2D). Proteins with the highest fold change related to cholesterol metabolism were NPC intracellular cholesterol transporter 2 (NPC2), NPC1, hydroxymethylglutaryl-CoA synthase (HMGCS1), apolipoprotein B receptor (APOBR), and sterol O-acyltransferase 1 (SOAT1) (Fig. 2E). In contrast, proteins linked to FA metabolism, such as cytochrome P450 2C44 (CYP2C23), acetyl-CoA carboxylase 2 (ACACB), CYP4A10, acyl-coenzyme A thioesterase 3 (ACOT3), and acetoacetyl-CoA synthetase (AACS), were found to be downregulated in the livers of *Lal−/−* mice, further highlighting the drastic metabolic shifts in these animals (Fig. 2G). Moreover, the livers of *Lal−/−* mice had 74 upregulated hydrolases, of which the five most upregulated enzymes (LYZ2, lactotransferrin (LTF), cathepsin S (CTSS), 5′-3′ exonuclease PLD3 (PLD3), napsin-A (NAPSA)) are not directly involved in metabolic processes but are associated with inflammation (Fig. 2G).

### Decreased lipid degradation and increased lipid accumulation in *Lal−/−* livers are independent of nutritional status

**Fig. 3:**
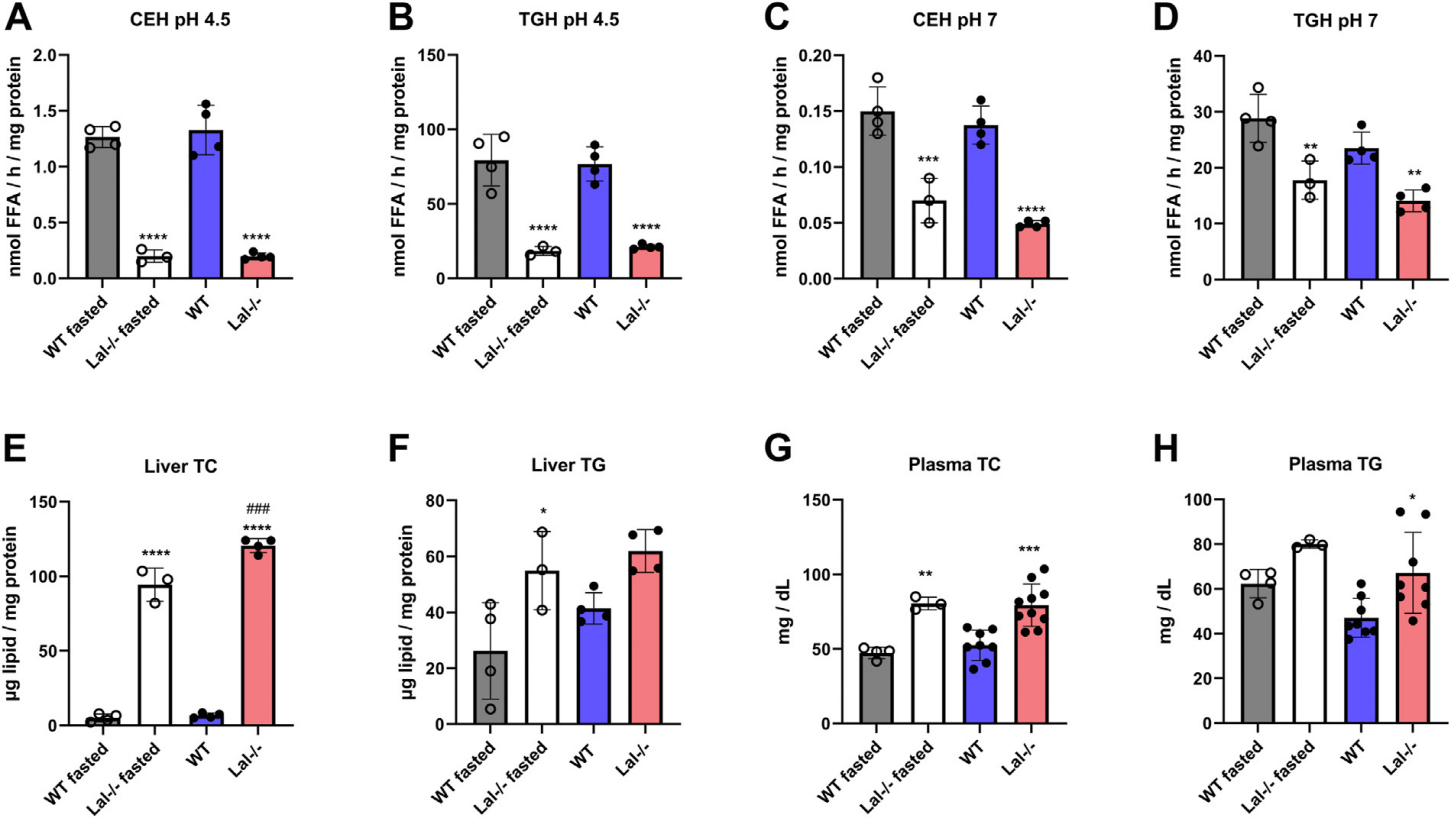
Impaired lipid hydrolysis in livers of *Lal−/−* mice. Liver tissue was isolated from WT and *Lal−/−* mice after 4 h of fasting and after an oral lipid bolus. Acid (pH 4.5) (A) cholesteryl ester hydrolase (CEH) and (B) triacylglycerol (TG) hydrolase (TGH) activities (n = 3–4), neutral (pH 7) (C) CEH and (D) TGH activities (n = 3–4), hepatic (E) total cholesterol (TC) and (F) TG concentrations (n = 3–4), and plasma (G) TC, and (H) TG concentrations (n = 3–10). Statistically significant differences were calculated by 2-way ANOVA followed by Tukey’s *post hoc* test. ∗*P* < 0.05, ∗∗*P* ≤ 0.01, ∗∗∗*P* ≤ 0.001, and ∗∗∗∗*P* ≤ 0.0001 for comparison between different genotypes within the same group (WT vs. *Lal−/−*, WT fasted vs. *Lal−/−* fasted). ^###^*P* ≤ 0.001 for comparison between the different conditions in the same genotypes (WT fasted vs. WT, *Lal−/−* fasted vs. *Lal−/−*). Data are presented as mean ± SD.

Given the observed proteome remodeling suggestive of reduced lipid metabolism processes, we further investigated the potential impairment of lipid degradation by measuring lipid hydrolase activities and lipid levels in the liver tissue of mice after 4 h of fasting and after an oral oil bolus, respectively. CE hydrolase (CEH) and TG hydrolase (TGH) activities at pH 4.5 were significantly decreased in the livers of *Lal−/−* compared to WT mice (Fig. 3A, B). This finding was expected since LAL is the only known enzyme responsible for CE and TG degradation at acidic pH (23). Intriguingly, CEH and TGH activities were also reduced at pH 7 in the livers of *Lal−/−* compared to WT mice (Fig. 3C, D), suggesting that LAL-D impairs lipid hydrolysis in both neutral and acidic pH environments. Consistent with the decreased lipase activities, hepatic TC and TG concentrations were markedly elevated in *Lal−/−* mice (Fig. 3E, F). Plasma TC and TG concentrations followed the same trend (Fig. 3G, H).

### LAL-D promotes liver inflammation

**Fig. 4:**
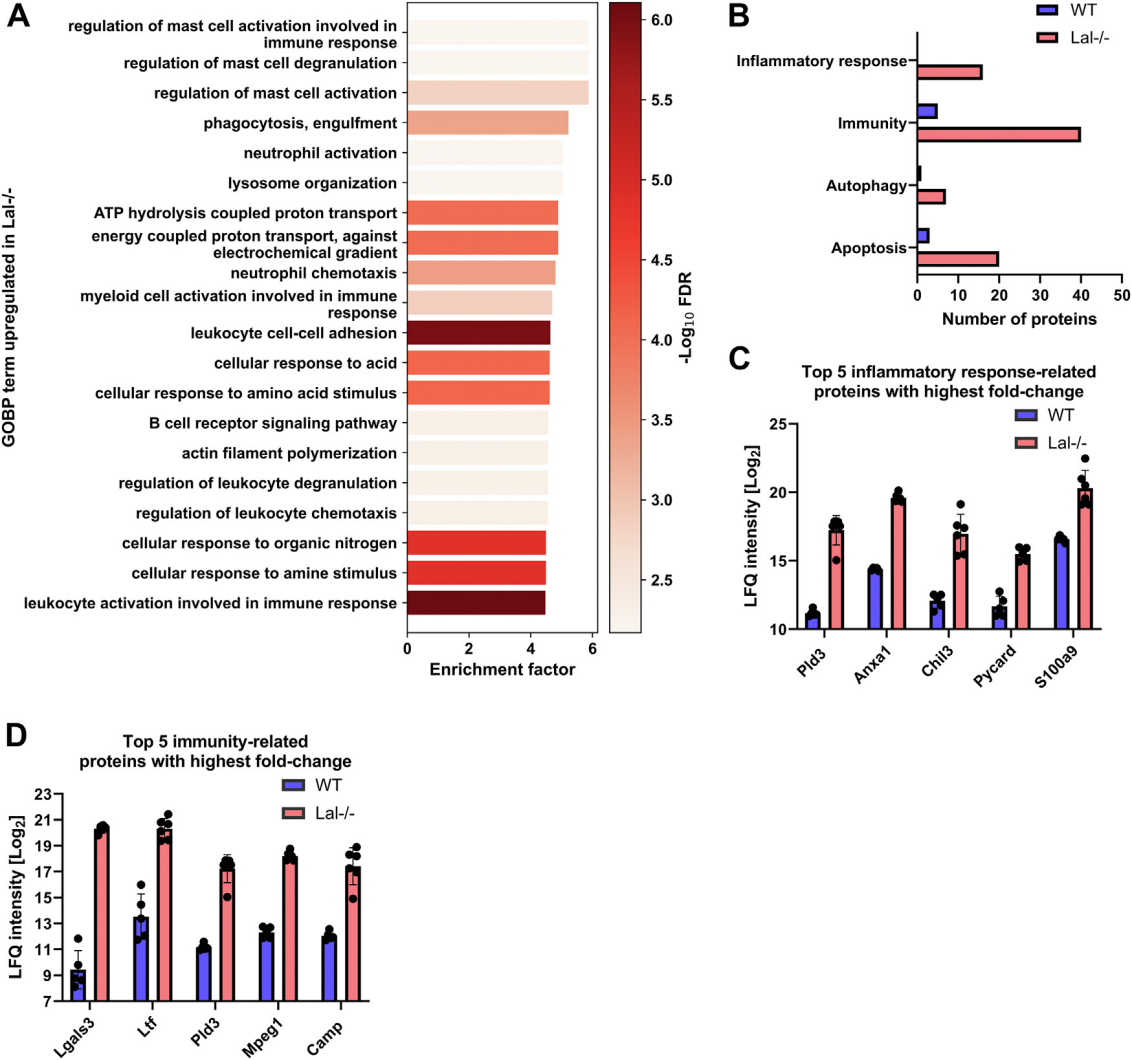
LAL deficiency is associated with pronounced liver inflammation. A: Top 20 gene ontology biological process (GOBP) terms upregulated in the liver of *Lal−/−* mice. B: Significantly changed proteins from WT and *Lal−/−* livers annotated to selected UniProt keyword terms. C: Top five proteins with the highest fold change annotated to the UniProt keyword *Inflammatory Response*. D: Top five proteins with the highest fold change annotated to the UniProt keyword *Immunity*. All figures represent data from 5 WT and 6 *Lal−/−* mice. Data are presented as mean or as mean ± SD.

We next focused on the 20 most enriched GOBP terms that were upregulated in the livers of *Lal−/−* mice. Consistent with the increased macrophage abundance in *Lal−/−* livers (13), GOBP terms of inflammation- and immune-related processes involving myeloid cells were highly enriched (Fig. 4A). In addition, the GOBP terms lysosome organization and ATP hydrolysis were among the strongest enriched terms (Fig. 4A), indicating impaired lysosomal function and energy generation. Given the observed enrichment in inflammation-related processes and lysosomal organization, we further investigated the extent to which significantly changed proteins were assigned to immunology-related UniProt keywords (Fig. 4B). Livers of *Lal−/−* mice displayed an increased number of upregulated proteins compared to WT mice in all UniProt terms examined, including inflammatory response, immunity, autophagy, and apoptosis. The top five proteins related to the inflammatory response that exhibited the highest fold change were PLD3, annexin A1 (ANXA1), chitinase-like protein 3 (CHIL3), apoptosis-associated speck-like protein containing a CARD (PYCARD), and protein S100-A9 (S100A9) (Fig. 4C). The highest upregulated protein related to immunity in the livers of *Lal−/−* mice was LGALS3 (also named MAC2), a well-known macrophage marker (Fig. 4D).

### LAL-D triggers comprehensive restructuring of the liver

**Fig. 5:**
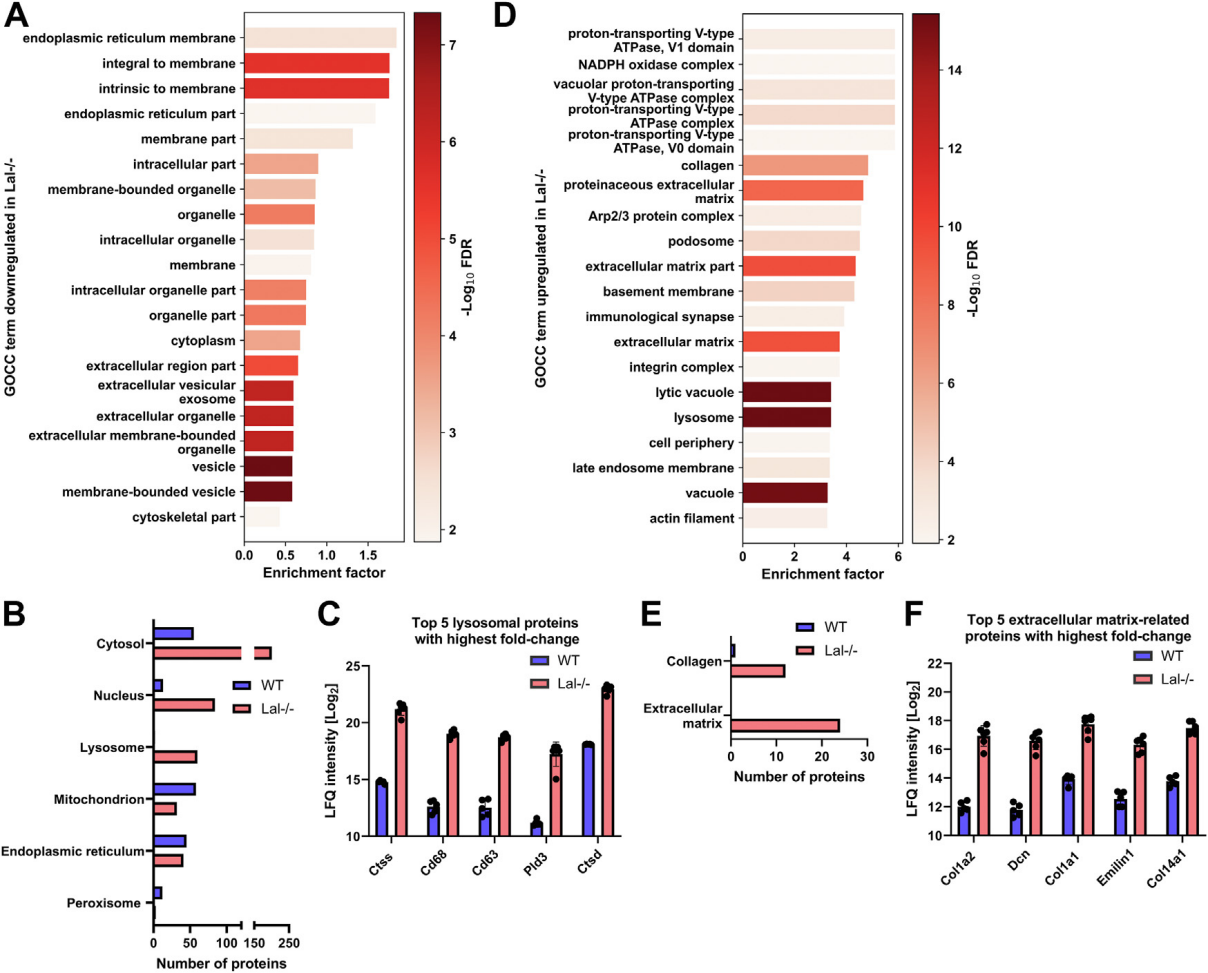
LAL deficiency causes major changes in liver cellular components. A: Top 20 gene ontology cellular component (GOCC) terms downregulated in livers of *Lal−/−* mice. B: Significantly changed proteins from WT and *Lal−/−* livers annotated to selected UniProt keyword terms representing various cellular compartments. C: Top five proteins with the highest fold change annotated to the UniProt keyword *Lysosome*. D: Top 20 GOCC terms upregulated in livers of *Lal−/−* mice. E: Significantly changed proteins from WT and *Lal−/−* livers annotated to selected UniProt keyword terms representing extracellular matrix. F: Top five proteins with the highest fold change annotated to the UniProt keyword *Extracellular Matrix*. All figures represent data from 5 WT and 6 *Lal−/−* mice. Data are presented as mean or as mean ± SD.

Since the livers of *Lal−/−* mice are markedly larger than WT livers (supplemental Fig. S1B), we examined how LAL-D affects the expression of proteins annotated to various cellular components. Among GOCC downregulated in *Lal−/−* livers, we observed multiple membrane and vesicular terms, suggesting a profound impact of LAL-D on membrane organization (Fig. 5A). By annotating significantly upregulated proteins to UniProt keywords representing different cellular compartments, we observed major differences between the livers of *Lal−/−* and WT mice (Fig. 5B). *Lal−/−* livers showed 58 upregulated lysosome-related proteins, including CTSS, macrosialin (CD68), CD63 antigen (CD63), PLD3, and CTSD (Fig. 5C). GOCC terms upregulated in the livers of *Lal−/−* mice revealed enrichment in multiple terms connected to ATP generation, indicating an increased energy requirement in LAL-D livers (Fig. 5D). Consistent with multiple upregulated GOCC terms indicative of extracellular matrix reorganization (Fig. 5D), we found an increased number of significantly upregulated proteins annotated to the UniProt keywords collagen and extracellular matrix in *Lal−/−* livers (Fig. 5E). Elevated expression levels of multiple extracellular matrix-related proteins, including collagen alpha-2(I) chain (COL1A2), decorin (DCN), COL1A1, elastin microfibril interface-located protein 1 (EMILIN1), and COL12A1, suggest increased fibrosis in livers of *Lal−/−* mice (Fig. 5F).

### Hepatocyte-specific LAL-D has a minor impact on the liver proteome

**Fig. 6:**
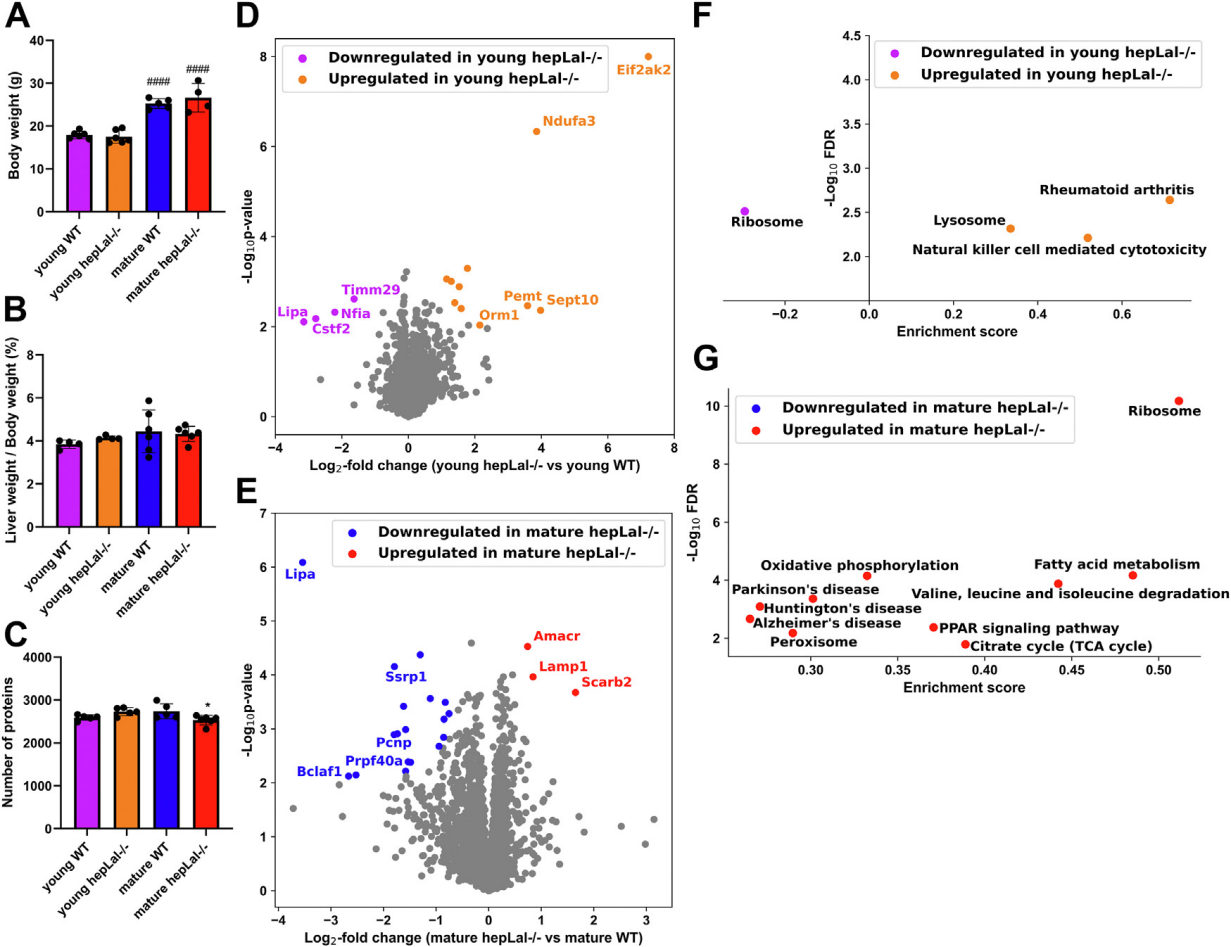
Minor proteome alterations in livers of young and mature hepatocyte-specific *Lal−/−* (*hepLal−/−*) mice. A: Body weight and (B) liver-to-body weight ratio of young (9–11 weeks of age) and mature (50–60 weeks of age) WT and *hepLal−/−* mice (n = 4–6). C: Quantified total proteome depth in livers from young (n = 5) and mature (n = 5–6) WT and *hepLal−/−* mice. D: Volcano plot of the liver proteome showing 11 significantly upregulated and four significantly downregulated proteins in young *hepLal−/−* mice (n = 5, FDR < 0.05, S0 = 0.1). E: Volcano plot of the liver proteome showing three significantly upregulated and 18 significantly downregulated proteins in mature *hepLal−/−* mice (n = 5–6, FDR < 0.05, S0 = 0.1). Kyoto encyclopedia of genes and genomes (KEGG) pathways enriched in livers from (F) young (n = 5) and (G) mature (n = 5–6) WT and *hepLal−/−* mice. Statistically significant differences for (A–C) were calculated by 2-way ANOVA followed by Tukey’s post-hoc test. ∗*P* < 0.05 for comparison between different genotypes within the same group (young WT vs. young *hepLal−/−*, mature WT vs. mature *hepLal−/−*). ^####^*P* ≤ 0.001 for comparison between the different conditions in the same genotypes (young WT vs. mature WT, young *hepLal−/−* vs. mature *hepLal−/−*). Data are presented as mean ± SD.

Since global LAL-D had a drastic effect on the liver proteome, we next tested whether hepatocyte-specific LAL-D comparably alters the liver proteome. For this purpose, we harvested livers from young (9–11 weeks of age) and mature (50–60 weeks of age) *hepLal−/−* mice. In contrast to high-fat/high-cholesterol diet-fed *hepLal−/−* mice with lower body and liver weight (15), body and liver weight were comparable between chow diet-fed *hepLal−/−* and WT mice (Fig. 6A, B). Despite reduced acidic CEH activity and a trend to decreased neutral CEH activity in the livers of young *hepLal−/−* mice (supplemental Fig. S2A, B), hepatic TC concentrations showed a non-significant trend toward increase in young and mature mice (supplemental Fig. S2C). Hepatic TG concentrations were unchanged (supplemental Fig. S2D), although acidic TGH activity was decreased in young *hepLal−/−* and WT mice; neutral TGH activity was comparable (supplemental Fig. S2E, F) between the genotypes. Plasma TC values were decreased in mature *hepLal−/−* mice (supplemental Fig. S2G), whereas plasma TG concentrations remained comparable in both genotypes during aging (supplemental Fig. S2H).

Employing an FDR of 0.01, we identified an average of 2,530–2,730 proteins in the analyzed livers (Fig. 6C). The quantified proteins covered approximately four orders of magnitude (supplemental Fig. S2I, J) and were normally distributed (supplemental Figs. S3A and S4A) in both young and mature mice. PCA of proteins detected in livers from young and mature *hepLal−/−* and WT mice showed a lower degree of clustering and separation (supplemental Figs. S3B and S4B) compared with the livers of *Lal−/− mice* (supplemental Fig. S1D). Of the 2,653 proteins quantified in the livers of young mice, 11 were upregulated and four were downregulated in *hepLal−/−* mice (Fig. 6D). The upregulated proteins included NADH dehydrogenase [ubiquinone] 1 alpha subcomplex subunit 3 (NDUFA3) and phosphatidylethanolamine N-methyltransferase (PEMT), indicating a minor alteration in oxidative phosphorylation and lipid processing. Of the 2,644 proteins quantified in the livers of mature mice, only three were significantly upregulated in *hepLal−/−* mice, including lysosome membrane protein 2 (SCARB2), lysosome-associated membrane glycoprotein 1 (LAMP1), and alpha-methylacyl-CoA racemase (AMCAR) (Fig. 6E). Since no significant KEGG pathway was found to be enriched by two-dimensional enrichment, we performed one-dimensional enrichment, resulting in several KEGG pathways being enriched in young *hepLal−/−* livers (e.g., rheumatoid arthritis, natural killer cell-mediated cytotoxicity, and lysosome) (Fig. 6F). *hepLal−/−* livers from mature mice showed enrichment in multiple KEGG pathways, with the most enriched pathways being ribosome, FA metabolism, valine, leucine, and isoleucine degradation, citrate cycle, and the PPAR signaling pathway (Fig. 6G).

## Discussion

Functional LAL activity is essential for life, and its partial or complete loss leads to a life-threatening lysosomal storage disease (7). Previous studies in mouse models have focused primarily on specific metabolic alterations resulting from LAL-D (14, 15, 16, 24, 25). To our knowledge, the present study is the first to provide a comprehensive analysis of the effects of global and hepatocyte-specific LAL-D on the liver proteome. These data will be of considerable benefit to researchers and clinicians focusing on LAL-D-related liver pathologies.

Significant changes in the liver proteome of *Lal−/−* mice, with the most dysregulated proteins related to metabolism or inflammation, coincided with the drastic remodeling of the liver, as evidenced by a more than threefold increase in the liver-to-body weight ratio. LGALS3, the highest upregulated protein in the livers of *Lal−/−* mice, is a well-known macrophage marker that plays a critical role in multiple liver pathologies and lysosomal repair (26), and its expression is upregulated in human liver fibrosis, regardless of etiology (27). Both LYZ2 (28) and CAPG (29) are predominantly expressed by macrophages and are essential for proper macrophage function (30, 31). These findings are consistent with the increased number of macrophages being observed in multiple organs of patients with LAL-D and mice (13, 32). GPNMB is implicated in immunosuppression and cancer progression (33), and UAP1L1 plays a critical role in the proliferation of hepatocellular carcinoma cells, whose knockdown significantly decreases human hepatoma cell proliferation (34). Upregulation of these proteins suggests that LAL-D increases hepatic expression of multiple cancer markers, which is in line with previous studies demonstrating that lack of LAL promotes tumor growth (35, 36, 37). In contrast, the most downregulated proteins in the livers of *Lal−/−* mice, MUP9 and MUP2, regulate glucose and lipid metabolism (38, 39). However, their importance in metabolic homeostasis was demonstrated only in male mice (39), and it is elusive whether the metabolic consequences of reduced MUPs expression in female *Lal−/−* mice may differ from those observed in males. Downregulation of CES3B may be associated with changes in lipoprotein metabolism and lipogenesis in *Lal−/−* livers. Inhibition of CES3B leads to reduced lipogenesis and increased fatty acid oxidation to protect the liver from high-fat diet-induced hepatic steatosis despite decreased VLDL secretion (40). This finding is consistent with reduced VLDL secretion and increased glucose utilization in *Lal−/−* mice (14). Downregulation of STAB2, a scavenger receptor involved in the uptake of oxidized LDL particles primarily being expressed in liver sinusoidal endothelial cells (41), implies that *Lal−/−* mice have impaired lipoprotein metabolism. Downregulation of PTPRD correlates inversely with NAFLD but directly with hepatocellular carcinoma development (42, 43). Along with numerous upregulated inflammation-related proteins, the downregulation in PTPRD and thus increased susceptibility to cancer development is consistent with previous studies demonstrating the important role of LAL in inflammation and immunosuppression (44, 45, 46). Collectively, the highest up- and downregulated proteins in *Lal−/−* livers demonstrate complex metabolic rewiring and increased inflammation.

Downregulated GOBP terms confirmed substantial changes in carbohydrate and lipid metabolic processes in the livers of *Lal−/−* mice. In line with previous studies showing increased glucose utilization and impaired cholesterol metabolism (13, 14), we also observed an upregulation in glycolysis- and cholesterol metabolism-related proteins in LAL-D livers. Altered lipid metabolism in *Lal−/−* mice was further supported by decreased liver lipid degradation and increased lipid accumulation. Interestingly, LAL-D mice also had reduced neutral lipase activities, possibly due to the markedly reduced amount of cytoplasmic lipid droplets and storage of lipids within the lysosomes (14). LAL-D-induced liver inflammation was evidenced by numerous upregulated inflammation-related GOBP terms. Mast cell activation and degranulation were among the highest upregulated GOBP terms. Interestingly, recent studies suggested that mast cells play a significant role in the progression of various liver diseases (47). The major proteins driving liver inflammation in *Lal−/−* mice included PLD3, ANXA1, and CHIL3. PLD3 and ANXA1 play important roles in the suppression of liver inflammation (48, 49). Treatment of mice with recombinant ANXA1 reduces liver inflammation and fibrosis without affecting steatosis and metabolism (50), suggesting a possible mechanism by which *Lal−/−* mice attempt to combat liver inflammation. Conversely, upregulated expression of CHIL3 has been demonstrated to actively promote liver fibrogenesis (51). In addition to increased inflammation and consistent with our previous data demonstrating decreased ATP levels in the livers of *Lal−/−* mice (14), the upregulated GOBP terms confirmed impaired lysosomal function and energy generation. The most enriched down- and upregulated GOCC terms further supported the pronounced liver reorganization in *Lal−/−* mice. Our results showed significant upregulation of multiple lysosomal proteins in *Lal−/−* livers, including CTSS and CTSD, which are known to be important mediators in liver fibrosis and liver cancer progression (52), along with CD68, a well-established macrophage marker (53). The upregulated GOCC also revealed enrichment of extracellular matrix-related proteins, similar to that observed in the liver proteome of genetically obese (*ob/ob*) mice (54). The most upregulated extracellular matrix proteins involved several collagens (COL1A2, COL1A1, and COL14A1), indicating extensive fibrosis in the livers of *Lal−/−* mice (55).

In contrast to global LAL-D, hepatocyte-specific LAL-D had only minor effects on the liver proteome. The mild phenotype of chow diet-fed *hepLal−/−* mice (15) might be due to elevated residual acidic CEH activity (40%) compared to the livers of global *Lal−/−* mice (15%). As one of the most upregulated proteins in the *hepLal−/−* livers was the known macrophage marker LGALS3, infiltrating enzyme-competent macrophages with high LAL expression may lead to the increased hepatic LAL activity in *hepLal−/−* compared with *Lal−/−* mice. Alternatively, LAL is a secreted enzyme that could be released from other cells and taken up by LAL-D hepatocytes, partially restoring activity and alleviating the condition. However, further studies are needed to investigate this phenomenon in more detail. Restoration of hepatic LAL activity by liver transplantation in patients with LAL-D often results in disease recurrence in the graft, probably because LAL activity is absent in infiltrating host macrophages (56). Among the few significantly changed proteins, the upregulation of PEMT in the livers of young *hepLal−/−* mice may indicate minor changes in lipid and energy metabolism (57). Conversely, upregulation of SCARB2 and LAMP1 in the livers of mature *hepLal−/−* mice suggests increased lysosome quantity and/or size (58, 59). Since the livers of *hepLal−/−* mice accumulate CE (15), the upregulation of SCARB2 and LAMP1 might result from an increased requirement for lysosomal export of cholesterol (58, 59). These data imply that only a minor change in lysosomal function occurs with advanced age in *hepLal−/−* mice. Interestingly, TC and TG levels in liver and plasma were comparable between WT and *hepLal−/−* mice at young and mature ages, which contrasts the animals fed a high-fat/high-cholesterol diet (15). Thus, future studies with this diet may be better suited to further investigate the consequences of hepatocyte-specific LAL-D. Concordantly, only a few KEGG pathways indicating dysregulation of lysosomal function were significantly enriched in young *hepLal−/−* livers, whereas mature *hepLal−/−* mice displayed enrichment in multiple KEGG pathways, suggesting a more profound impact of hepatic LAL-D on metabolism and signaling at older ages.

In summary, our findings highlight the drastic consequences of global LAL-D on the liver proteome, with alterations in lipid and glucose metabolism, inflammation, and liver function. In contrast, hepatocyte-specific LAL-D caused only minor changes in the liver proteome, suggesting that LAL from other liver cell types may be able to compensate for the loss of LAL in hepatocytes when mice are not challenged with HF/HCD.

## Data availability

The mass spectrometry proteomics datasets have been deposited to the ProteomeXchange Consortium via the PRIDE partner repository (60) with the dataset identifier PXD042478.

## Supplemental data

This article contains supplemental data.

## Conflict of interest

The authors declare that they have no known competing financial interests or personal relationships that could have appeared to influence the work reported in this paper.
