# Supplementary material for: Metabolic changes and propensity for inflammation, fibrosis, and cancer in livers of mice lacking lysosomal acid lipase
Source: J Lipid Res. 2023 Aug 16;64(9):100427. doi: 10.1016/j.jlr.2023.100427 (PMC10482749; doi:10.1016/j.jlr.2023.100427)
Supplement: Supplemental Figures S1–S4 [file mmc1.pdf]

Supplemental figures

Metabolic changes and propensity for inflammation, fibrosis, and cancer in livers of mice lacking lysosomal acid lipase

Ivan Bradić, Laura Liesinger, Katharina B. Kuentzel, Nemanja Vujić, Michael Trauner, Ruth Birner-Gruenberger, Dagmar Kratky

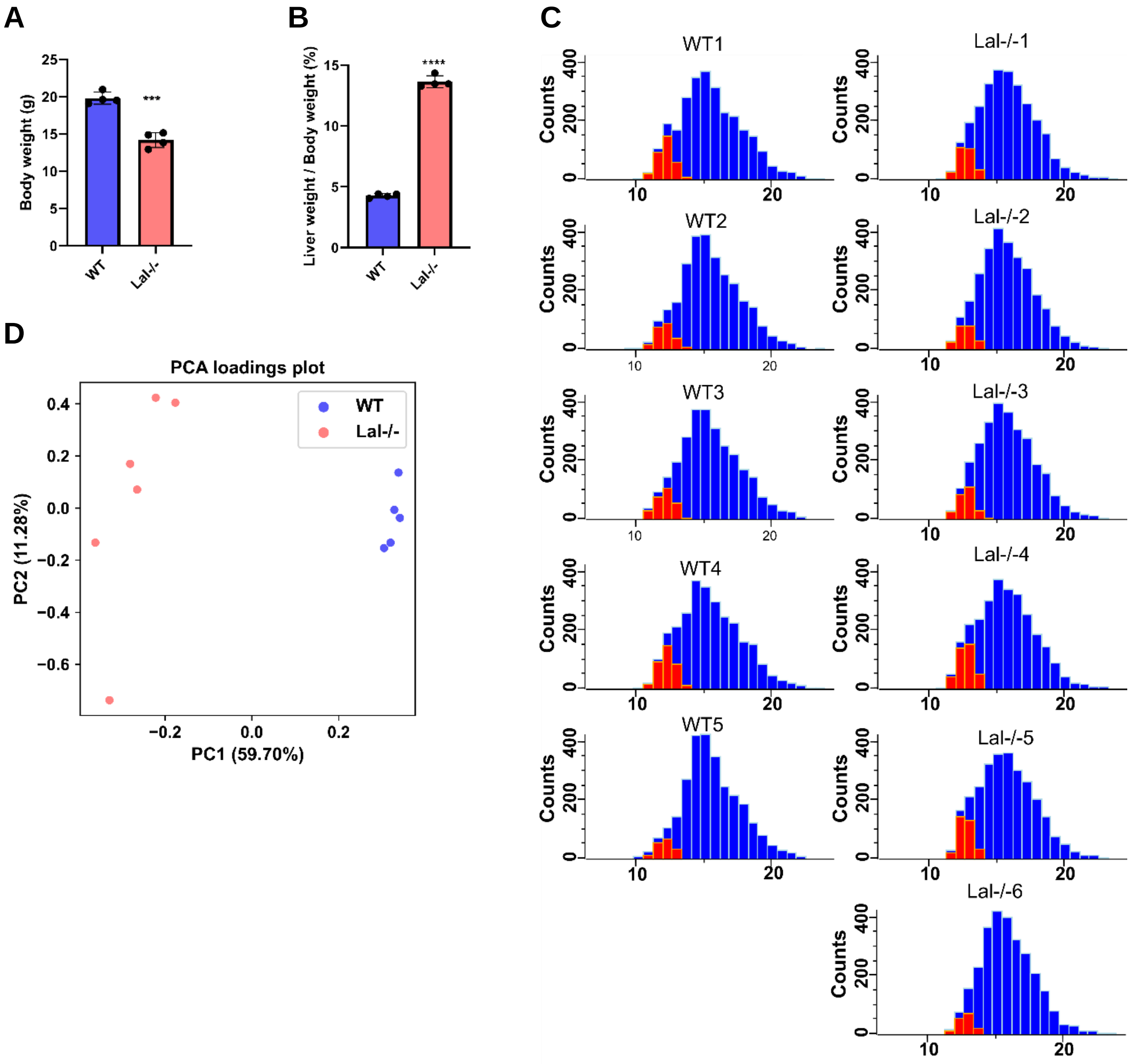

Figure S1. Changes in liver and body weight in *Lal-/-* mice and quality control of proteomics data. (A) Body weight and **(B)** liver-to-body weight ratio of WT and *Lal-/-* mice (n = 4). **(C)** Histograms of proteomics data from WT and *Lal-/-* mice livers with imputed values shown in red. **(D)** Principal component analysis (PCA) plot of liver proteome data from WT and *Lal-/-* mice. Statistically significant differences for **(A, B)** were calculated by Student's t-test. ***p ≤ 0.001, ****p ≤ 0.0001. Data are presented as mean ± SD.

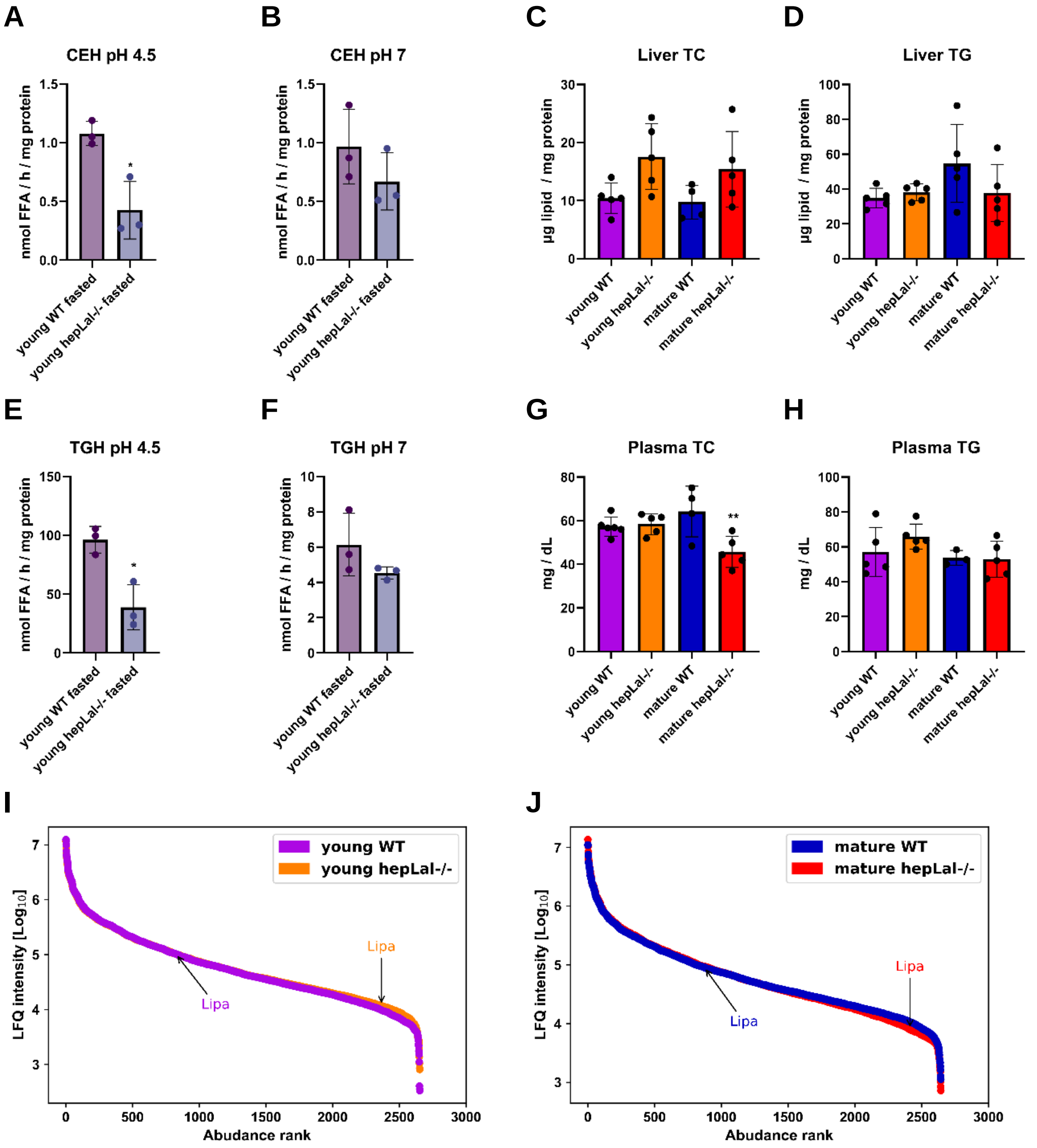

Figure S2. Liver and plasma lipids and dynamic proteome range in livers of young and mature hepatocyte-specific *Lal-/-* (*hepLal-/-*) mice. Liver **(A)** acid (pH 4.5) and **(B)** neutral (pH 7) cholesteryl ester hydrolase (CEH) activities (n = 3) in young WT and *hepLal-/-* mice. Liver **(C)** total cholesterol (TC) and **(D)** triacylglycerol (TG) concentrations from young (9 -11 weeks of age) and mature (50-60 weeks of age) WT and *hepLal-/-* mice (n = 4 – 5). Liver **(E)** acid and **(F)** neutral triacylglycerol hydrolase (TGH) activities (n = 3**)**. Plasma **(G)** TC and **(H)** TG concentrations (n = 3 – 6). Dynamic range of the liver proteome from **(I)** young (n = 5) and **(J)** mature WT and *hepLal-/-* mice (n = 5 – 6) based on $\log_{10}$ of the mean intensity of LFQ ordered by the rank of abundance. **(A, B, E, F)** Statistically significant differences were calculated by Student's t-test. Data are presented as mean ± SD. **(C**, **D**, **G**, **H)** Statistically significant differences were calculated by 2-way ANOVA followed by Tukey's post-hoc test. * $p < 0.05$, ** $p \leq 0.01$ for comparison between

different genotypes within the same group (young WT vs young *hepLal-/-*, mature WT vs mature *hepLal-/-*). Data are presented as mean ± SD.

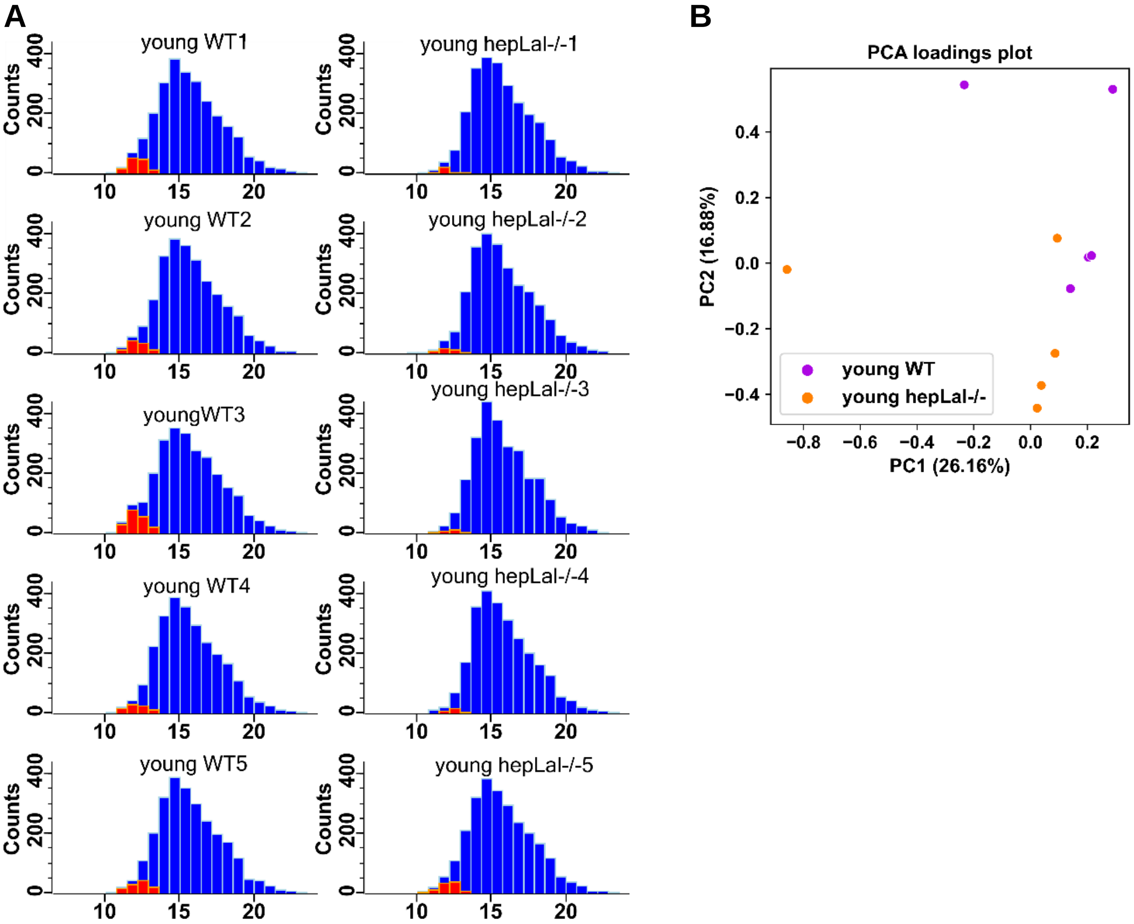

Figure S3. Quality control of liver proteomics data from young WT and *hepLal-/-* mice. (A) Histograms of proteomics data from young WT and *hepLal-/-* mice livers with imputed values shown in red (n = 5). **(B)** Principal component analysis (PCA) plot of the liver proteome from young WT and *hepLal-/-* mice (n = 5).

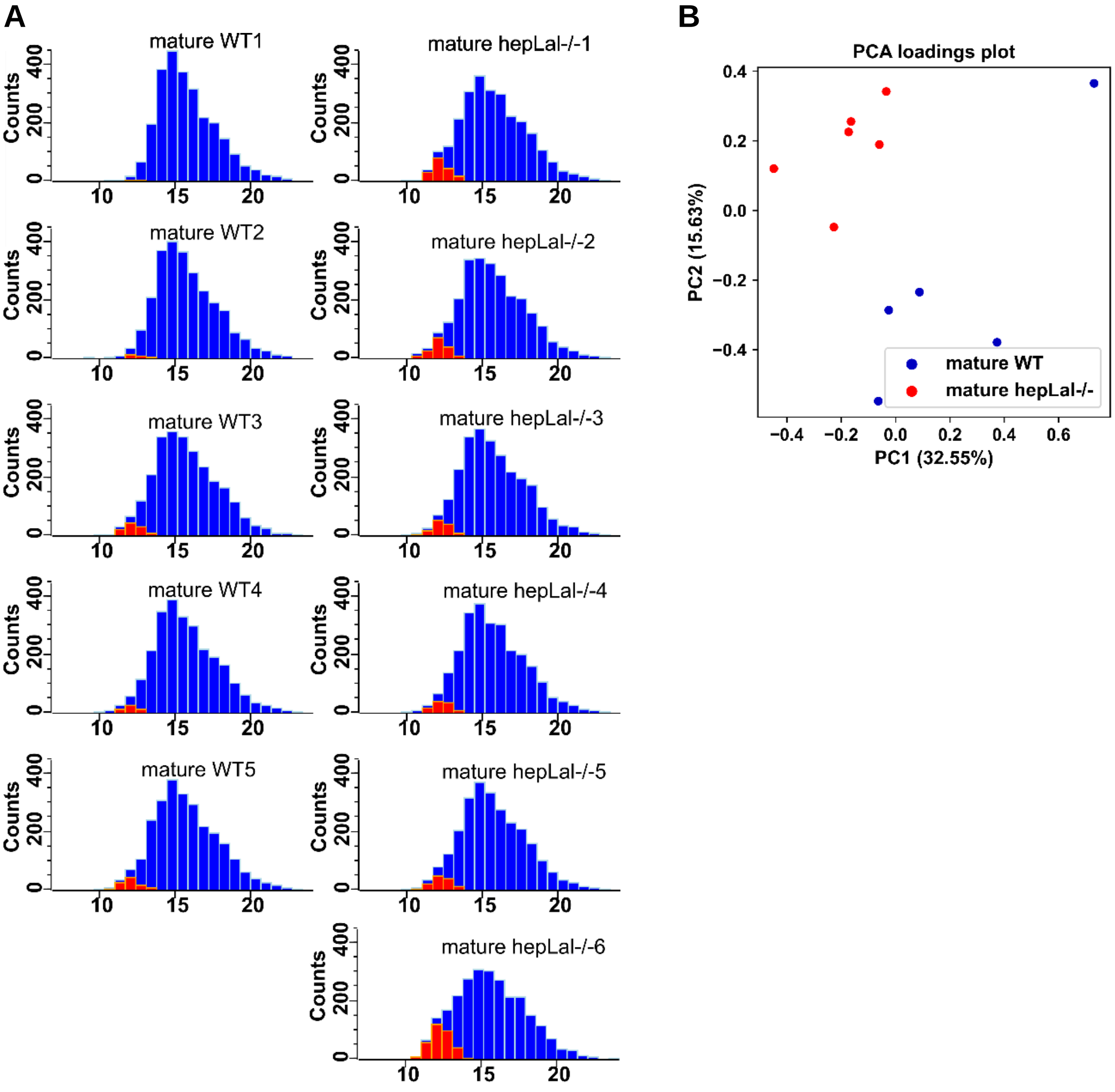

Figure S4. Quality control of liver proteomics data from mature WT and *hepLal-/-* mice. (A) Histograms from mature WT and *hepLal-/-* mice liver proteome with imputed values showed in red (n = 5 – 6). **(B)** Principal component analysis (PCA) plot of the liver proteome from mature WT and *hepLal-/-* mice (n = 5 – 6).
